# Navigating Metabolic Challenges in Ovarian Cancer: Insights and Innovations in Drug Repurposing

**DOI:** 10.1002/cam4.70681

**Published:** 2025-02-19

**Authors:** Sara Mikhael, Georges Daoud

**Affiliations:** ^1^ Department of Anatomy, Cell Biology and Physiological Sciences, Faculty of Medicine American University of Beirut Beirut Lebanon

**Keywords:** drug repurposing strategy, metabolic reprogramming, metabolic syndrome, ovarian cancer

## Abstract

**Background:**

Ovarian cancer (OC) is the most lethal gynecological malignancy and a major global health concern, often diagnosed at advanced stages with poor survival rates. Despite advancements in treatment, resistance to standard chemotherapy remains a critical challenge with limited treatment options available. In recent years, the role of metabolic reprogramming in OC has emerged as a key factor driving tumor progression, therapy resistance, and poor clinical outcomes.

**Methods:**

This review explores the intricate connections between metabolic syndrome, enhanced glycolysis, and altered lipid metabolism within OC cells, which fuel the aggressive nature of the disease. We discuss how metabolic pathways are rewired in OC to support uncontrolled cell proliferation, survival under hypoxic conditions, and evasion of cell death mechanisms, positioning metabolic alterations as central to disease progression. The review also highlights the potential of repurposed metabolic‐targeting drugs, such as metformin and statins, which have shown promise in preclinical studies for their ability to disrupt these altered metabolic pathways.

**Conclusion:**

Drug repurposing offers a promising strategy to overcome chemoresistance and improve patient outcomes. Future research should focus on unraveling the complex metabolic networks in OC to develop innovative, targeted therapies that can enhance treatment efficacy and patient survival.

## 1 Ovarian Cancer Overview

When fatal malignancies in women come to mind, one inherently thinks of breast cancer. Pink ribbons fill the month of October, packed with awareness campaigns, posters, flyers, rallies, and screening procedures. Nevertheless, Ovarian Cancer (OC) stands as the most lethal gynecological malignancy, ranking among the top five leading causes of cancer‐related deaths in women globally [1, 2]. OC diagnosis predominantly relies on the pathological analysis of a biopsy [3]. It exhibits diverse genetic patterns and substantial phenotypic heterogeneity [4]. According to the 5th edition of the WHO guidelines updated in 2020, Ovarian Neoplasms are classified as Epithelial tumors, Mesenchymal tumors, Mixed epithelial and mesenchymal tumors, Germ cell tumors, sex‐cord stromal tumors, miscellaneous tumors, tumor‐like lesions, and Secondary tumors. This classification closely aligns with the 4th edition [5, 6].

The prognosis of OC is intricately tied to various factors, with the stage at initial diagnosis being a critical determinant. If OC is diagnosed at an early stage where the tumor is restricted to the ovaries (Stage I), the 5‐year OS reaches around 90% [2, 7]. Progression to Stage II, characterized by tumor extension to the pelvic region, is associated with a 5‐year OS rate of 70%. However, the survival rate drops sharply to 20% if OC is diagnosed in stages III or IV, where the disease has advanced beyond the pelvic area. However, even with advancements in treatment modalities, the emergence of treatment resistance poses a significant challenge to prognosis. OC is often characterized by resistance to standard platinum‐based chemotherapy, contributing to the complexity of managing the disease. The development of chemoresistance can lead to a recurrence of the disease, impacting overall survival rates [7].

## 2 Challenges in Therapy

Research efforts continue to explore early detection methods, innovative therapies, and a deeper understanding of the molecular underpinnings of OC to enhance prognostic accuracy and therapeutic options for OC patients.

### 2.1 Metabolic Syndrome

Numerous factors contribute to either elevating or diminishing the risk of OC as well as complicating the treatment journey. The Metabolic Syndrome (MetS) is a collective term for a several risk factors associated with obesity, diabetes mellitus (DM) and cardiovascular diseases, with insulin resistance serving as a key distinguishing characteristic. Furthermore, another fundamental property is chronic proinflammation [8]. Notably, a recent study by Watanabe et al. [9] revealed that individuals with metabolic syndrome had a 33% higher cancer‐related mortality rate compared to those without metabolic conditions. Moreover, the mortality rate increased with the number of MetS components the patient exhibited.

#### 2.1.1 Obesity

Several studies have investigated the link between obesity and OC. In a Mendelian randomized study, analyzing data from 39 studies, it was observed that a higher Body Mass Index (BMI) was associated with an increased risk specifically for low‐grade serous cancers and not with other subtypes [10]. A meta‐analysis including findings from 26 observational studies further emphasized that the risk effect escalated with higher levels of obesity. Additionally, the significance of the risk was influenced by age, with a statistically significant association observed in early life and premenopausal obesity rather than in the postmenopausal period [11]. In addition to its role as a risk factor, obesity not only affects the risk of developing OC but also has implications for the prognosis of the disease. Obese women diagnosed with OC face greater challenges in their treatment journey and have poorer prognoses compared to their non‐obese counterparts. One key aspect contributing to this disparity is the altered tumor microenvironment in obese individuals, characterized by elevated levels of adipose tissue‐derived factors and hormones. Excess adiposity in obese individuals leads to dysregulated production of adipokines, cytokines, and growth factors, creating a pro‐inflammatory and pro‐tumorigenic milieu within the tumor microenvironment. Adipokines such as leptin and adiponectin, which are normally involved in regulating metabolism and inflammation, are aberrantly secreted in obesity and can directly influence OC progression through various signaling pathways. Moreover, chronic inflammation associated with obesity further exacerbates tumor growth and metastasis by promoting angiogenesis, immune evasion, and resistance to therapy [12].

Furthermore, obesity is intricately linked with metabolic dysregulation, including insulin resistance and hyperinsulinemia, which contribute to the development and progression of OC. Insulin and insulin‐like growth factor‐1 (IGF‐1), which are elevated in obese individuals, act as potent mitogens and survival factors for cancer cells, stimulating proliferation, inhibiting apoptosis, and enhancing tumor cell migration and invasion. Other hormones dysregulated include elevated estrogen levels, influenced by obesity, which contribute to the heightened proliferation of ovarian epithelial cells, particularly notable in the endometrioid subtype of OC [13, 14]. Additionally, increased androgen levels, associated with obesity, may elevate the risk of ovarian cancer, as seen in conditions such as polycystic ovarian syndrome [14]. These metabolic alterations not only fuel tumor growth but also confer resistance to chemotherapy and targeted therapies, posing significant challenges in the management of OC in obese patients.

#### 2.1.2 Diabetes Mellitus

The relationship between DM and cancer is intricate and multifaceted. A study by Bakhru et al. [15] examined and compared clinical outcomes between 570 patients with primary peritoneal, ovarian, and fallopian tube cancer who were not diabetic and 72 individuals who were diabetic for over a decade. Diabetic ovarian cancer patients exhibited higher body mass indexes (BMIs) and a greater prevalence of chronic, comorbid illnesses. The median overall survival (OS) for diabetic patients across a 10‐year span was 1503 days, while non‐diabetic patients had a longer median OS of 2464 days. This highlights that OC patients with diabetes as a chronic condition experienced a significantly worse survival rate than those without diabetes.

From a molecular viewpoint, elevated levels of IGF‐1, insulin, cytokines, and estrogen collectively contribute to a heightened risk of malignancy [12]. Insulin resistance is considered the most essential factor underlying the link between obesity, hyperglycemia, and carcinogenesis [8]. Hyperinsulinemia resulting from insulin resistance and subsequent hyperglycemia promotes cancer development by raising circulating levels of free IGF‐1 and IGF‐2, as reported in OC [16]. A study by Spentzos et al. [17] performed microarray profiling on primary ovarian tumor tissues from patients with advanced disease to fully assess the gene expression profiles of the entire IGF axis. Results showed a negative correlation between the survival of EOC and the simultaneous rise in IGF‐1 and IGFR. Insulin and IGF can activate the transcription factor, hypoxia‐inducible factor 1‐alpha (HIF‐1), implicated in a cascade of signaling pathways regulating cellular metabolism, apoptosis, invasion, and metastasis of malignant cells [8, 12]. Another important component of this relationship is hyperglycemia, the most evident clinical sign of DM, which stimulates tumor growth through pathways involving proliferation, anti‐apoptosis, migration, and metastasis [18, 19].

#### 2.1.3 Dyslipidemia

Increased levels of triglycerides, LDL cholesterol, and diminished HDL cholesterol function as risk factors in gynecological cancers. In a recent nested case–control study, Zeleznik et al. [20] investigated the relationship between circulating metabolites and OC. The findings reveal a negative correlation between OC risk and levels of sphingolipids, total cholesterol, and triacylglycerol.

### 2.2 Metabolic Reprogramming

**FIGURE 1:**
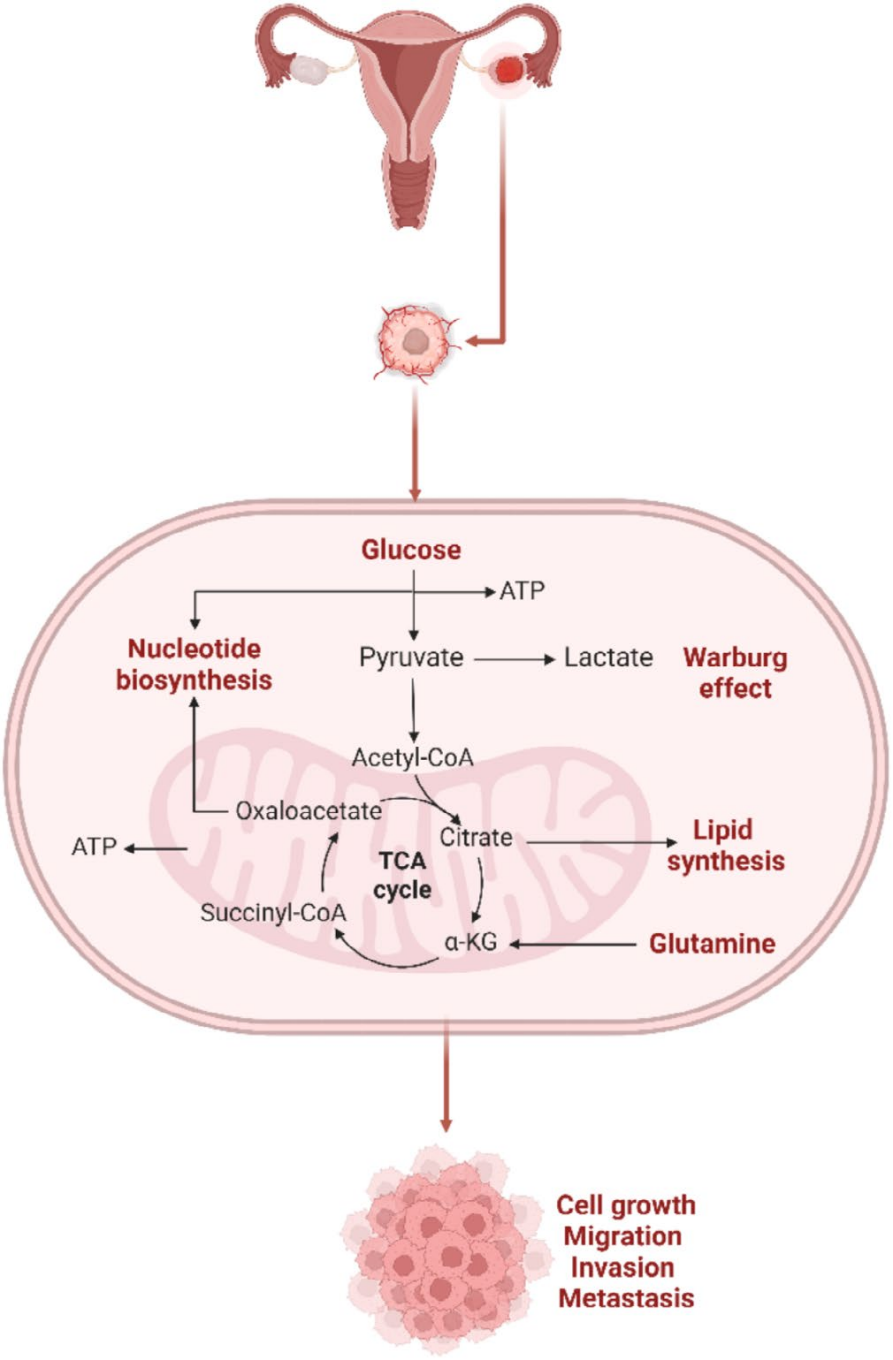
The interaction between OC cells with metabolic reprogramming. The Warburg effect occurs when most cancer cells, including OC, increase glucose intake and lactate generation. Aerobic glycolysis, glutamine catabolism, and lipid and nucleotide synthesis all contribute to OC cell proliferation, migration, invasion, and metastasis and are potential therapeutic targets in OC therapy.

It is now evident that cancer is characterized by inherent metabolic reprogramming, among other cellular hallmarks including uncontrolled proliferation, resisting growth‐suppressing signaling, overlooking apoptotic signals, self‐sufficiency, immortality, enhanced angiogenesis, evading the immune system, stimulating invasion and metastasis, and occurring in tumors driven by distinct signaling pathways across diverse tissues. Yet, a critical question arises: is metabolic reprogramming a causative factor or a consequence of the transformation process? In this section, our focus centers on examining metabolism as an initiator of OC through genetic and non‐genetic alterations, exploring the key pathways and molecular players while further posing challenges on OC therapy (Figure 1).

#### 2.2.1 Overview

The initiation of oncogenic activation was initially demonstrated to induce metabolic reprogramming. Subsequently, it was shown that loss‐of‐function mutations in metabolic enzymes contribute to the etiology of various hereditary cancers, including ovarian and breast cancer. These findings not only suggest a causal role of metabolic rewiring in cancer development but also challenge the conventional notion that it is merely a consequence of malignancy [21].

In the context of OC, it is not considered a singular disease but rather a heterogeneous group of malignancies with diverse genetic and molecular profiles. This heterogeneity poses a challenge in developing targeted therapies that can effectively address the specific characteristics of each subtype. Therefore, there exists a critical need to deepen our understanding of how cancerous cells strategically rewire their metabolism. This understanding is imperative to counteracting nutrient scarcity challenges and overcoming resistance to therapeutic interventions. Researchers are actively exploring targeted therapies that disrupt specific metabolic pathways to enhance the efficacy of OC treatments [22, 23].

#### 2.2.2 Glucose Metabolism and the Warburg Effect

Tumor metabolic reprogramming is a complex process intricately linked to cellular respiration, particularly glucose metabolism, which plays a pivotal role in meeting the escalating demands of cellular proliferation. This adaptive response allows the cancer cell to obtain crucial intermediary molecules, including nucleotides, lipids, and amino acids. These bioactive molecules serve as essential building blocks for an array of metabolic and non‐metabolic pathways that collectively drive the relentless growth and expansion of OC cells. Dr. Otto Warburg's groundbreaking research uncovered a fundamental alteration in energy metabolism, showcasing increased glucose uptake in tumors compared to normal cells (161). Glucose, a crucial ATP precursor, plays a central role in cellular energy synthesis, particularly in cancer cells [24].

Under normal physiological circumstances, oxidative phosphorylation (OXPHOS), involving the coupling of oxidation reactions with the mitochondrial electron transport chain (ETC), stands as the most efficient method for ATP production. Glucose undergoes glycolysis, producing pyruvate that enters the Krebs cycle, fueling OXPHOS and ATP production. However, in hypoxic conditions, where oxygen availability is limited, glycolysis becomes the exclusive means of providing cellular energy, as pyruvic acid is converted to lactic acid through anaerobic glycolysis. Despite the faster ATP production rate of glycolysis, its efficiency is lower than that of OXPHOS, which yields 32 to 36 ATP molecules from one glucose molecule [24, 25, 26].

**FIGURE 2:**
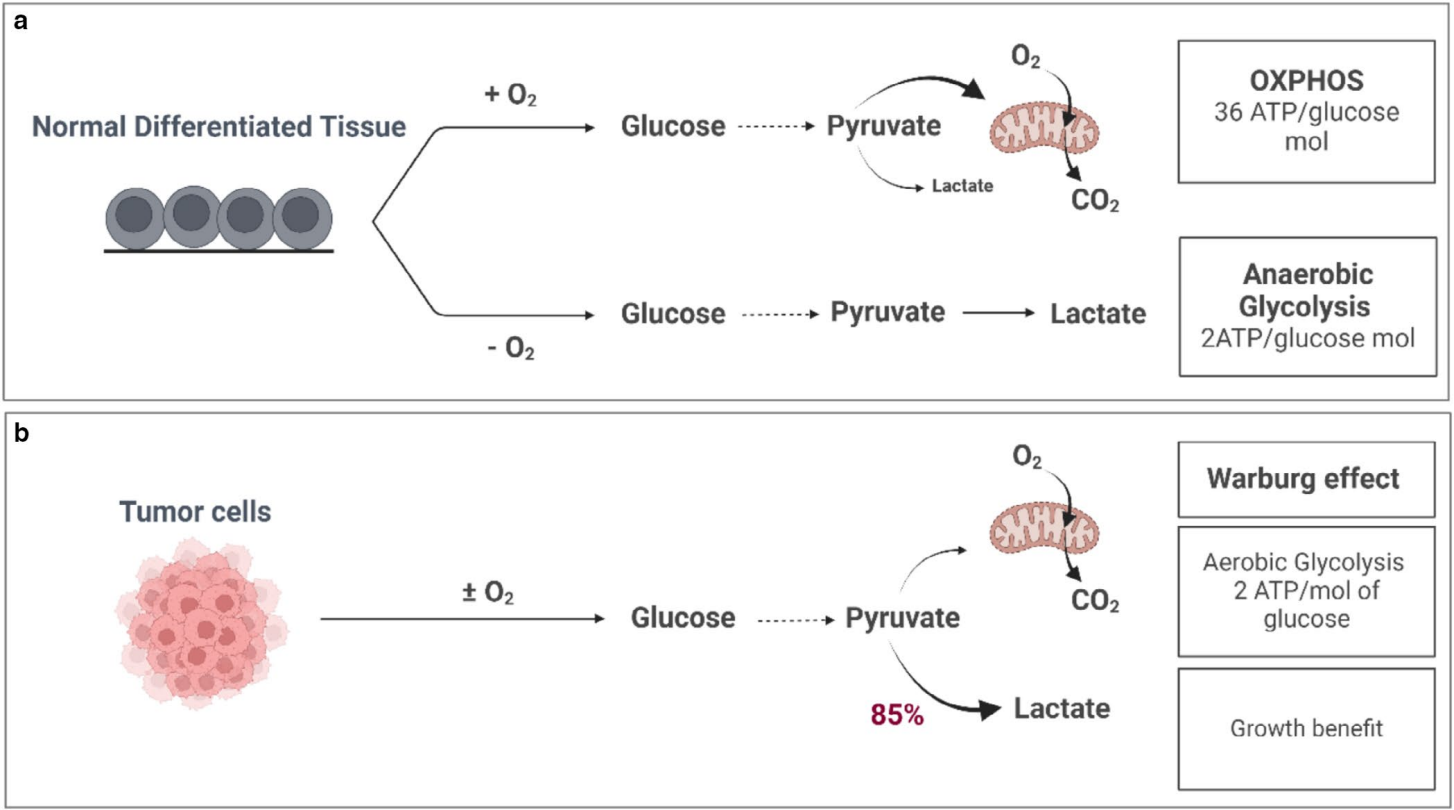
Glucose metabolism between normal differentiated tissue and tumor cells. (a) In normal differentiated tissues, glucose is processed through one of two pathways. In the presence of oxygen, glucose is metabolized into pyruvate, entering oxidative phosphorylation (OXPHOS) to generate approximately 36 mol ATP/mol glucose. In the absence of oxygen, glucose is converted to lactate, yielding 2 mol ATP/mol glucose. (b) Tumors and highly proliferative cells predominantly channel their glucose toward lactate production, resulting in approximately 4 mol ATP/mol glucose, even when oxygen is available. This phenomenon, known as the Warburg Effect, leads to a faster chemical reaction compared to OXPHOS, providing a significant growth advantage to cancer cells, despite producing less ATP/mol glucose. ATP: Adenosine triphosphate; CO_2_: Carbon dioxide; O_2_: oxygen; mol: mole.

Non‐tumoral cells can temporarily shift to fatty acid oxidation (FAO) during low glucose conditions, such as fasting [23]. However, cancer cells, including OC cells, exhibit a distinct metabolic profile with a preference for glycolytic metabolism, even in oxygen‐rich environments, a phenomenon known as the Warburg effect (Figure 2) [27, 28]. Even though the catabolism of glucose into lactate yields less ATP yield but it ensures rapid energy demand and provides intermediates for anabolic reactions [24, 29]. Subsequently, to satisfy the cell's high anabolic demands with low energy yields, glucose consumption automatically increases [30]. This phenomenon is portrayed by the increased glucose transporters to enhance the absorption of glucose molecules, increased glycolytic enzymes and regulators, as well as an upregulation of downstream pathways activated by the processes of glycolysis [31]. The Warburg effect underscores the adaptability of cancer cells to prioritize glycolysis for sustained growth and proliferation.

#### 2.2.3 Lipid Metabolic Reprogramming

The metabolic landscape in OC exhibits heterogeneity, as the survival of cancer cells depends not only on intracellular metabolism but also on the surrounding microenvironment [32]. Increasing evidence suggests that alongside altered glucose metabolism, OC entails dysregulated fatty acid and lipid metabolism, crucial for supporting cancerous cell proliferation and progression [33, 34, 35, 36]. Lipid metabolism is strictly regulated by rate‐limiting enzymes and is strongly correlated with glucose metabolism [37]. Lipids, comprising a diverse array of compounds, serve as essential fuel sources and bioactive components in various cellular processes such as inflammation, immunity, and cell differentiation [37, 38, 39]. These lipids are transported and utilized within cells through uptake, lipogenesis, usage, and storage mechanisms [37]. Notably, in OC, which frequently metastasizes to the omentum, a lipid‐rich environment, cancer cells often adopt alternative lipid‐dominant metabolic pathways. The omentum, a fatty pad surrounding the intestine, acts as an endocrine organ and a storage place for energy‐dense lipids [32]. Aberrant expression of numerous lipid metabolic enzymes in OC cells may facilitate metabolic pathway rewiring, contributing to lipid synthesis or breakdown mechanisms that provide essential building blocks and energy for malignant cellular proliferation and development [38].

##### 2.2.3.1 Activation of Lipogenesis

The term lipogenesis corresponds to the synthesis pathway of fatty acid as well as the synthesis of cholesterol through the mevalonate pathway [38].

###### 2.2.3.1.1 Fatty Acid Synthesis

The study of fatty acid metabolism in the context of cancer growth and metastasis unveils a complex interplay of metabolic adaptations crucial for tumor survival and progression. Unlike normal cells, cancer cells often exhibit heightened rates of de novo fatty acid biosynthesis, prioritizing endogenous synthesis over reliance on exogenous dietary lipids. This metabolic shift serves as a strategic response to the hostile tumor microenvironment characterized by hypoxia and nutrient deprivation, providing cancer cells with a survival advantage [32].

Fatty acid synthesis originates from cytoplasmic acetyl‐CoA, primarily derived from citrate generated via glucose, glutamine, or acetate metabolism. ATP‐citrate lyase (ACLY) catalyzes the conversion of citrate to acetyl‐CoA and oxaloacetate, with subsequent carboxylation of acetyl‐CoA yielding malonyl‐CoA. These precursor molecules fuel the enzymatic cascade orchestrated by Fatty Acid Synthase (FASN), culminating in the production of saturated fatty acids, notably palmitate [40]. Following its synthesis, palmitate undergoes elongation and desaturation processes, yielding a diverse cellular reservoir of non‐essential fatty acids contributing to the biosynthesis of crucial lipid molecules, including cholesterol, eicosanoids, and prostaglandins [41].

Altered expression of enzymes involved in fatty acid synthesis, such as acetyl‐coA carboxylase (ACC), FASN, and stearoyl‐CoA desaturase 1 (SCD1), is observed in OC cells, associated with aggressive tumor behavior and poor prognosis [23, 41, 42, 43]. Sterol Regulatory Element‐Binding Proteins (SREBPs) regulate the expression of these enzymes, with SREBP‐1 enhancing fatty acid synthesis gene expression. Additionally, the activation of SREBPs is subject to regulation by upstream oncogenic signaling pathways, notably the PI3K/Akt/mTORC1 signaling axis [41].

###### 2.2.3.1.2 Cholesterol de Novo Biosynthesis—Mevalonate Pathway

Extensive research has been conducted on the reprogramming of the mevalonate pathway (MVA) in cancer, particularly in regard to the synthesis of vital lipids like cholesterol, vitamin D, and lipoproteins. Cholesterol, a pivotal metabolite, orchestrates crucial modifications in cellular signaling pathways governing survival, proliferation, immune response, and inflammation, while also ensuring the fluidity and structural integrity of cell membranes [32, 41]. Human cholesterol metabolism is a multifaceted process, with cholesterol being acquired exogenously from the diet or synthesized endogenously, the latter contributing to approximately 70% of total body cholesterol content [44]. The pathways for intracellular cholesterol metabolism include de novo cholesterol synthesis, exogenous uptake, storage, and efflux [32, 44].

The intricate process of cholesterol biosynthesis occurs widely in mammalian cells, primarily in the liver and gut, through the MVA pathway involving over 20 enzymes. This pathway starts with acetyl‐CoA and produces various essential molecules, including cholesterol, steroid hormones, and bile acids, critical for cellular metabolism. Key enzymes such as HMG‐CoA reductase (HMGCR), farnesyl diphosphate farnesyltransferase 1 (FDFT1), and squalene epoxidase (SQLE) regulate rate‐limiting steps in this pathway [32, 44, 45]. Numerous enzymes within the MVA are frequently upregulated in cancer, including HMGCR, farnesyl diphosphate synthase (FDPS), geranylgeranyl pyrophosphate synthase (GGPPS), squalene synthase (SS), and SQLE. Transcriptional regulation of these enzymes is governed by SREBPs, notably SREBP2, which preferentially regulates genes involved in MVA and cholesterol biosynthesis. Activation of SREBP2, mediated by the PI3K/Akt/mTORC1 signaling axis, leads to heightened expression of HMGCR, increasing metabolic flux through the MVA pathway [41, 44, 45]. Furthermore, SREBP2 can engage in interactions with mutant p53, facilitating post‐translational modifications of oncogenes, such as Ras, and regulating mediators of epigenetic alterations, including histone deacetylases (HDACs) and DNA methyltransferases (DNMTs) [46]. The increased expression of HMGCR in cancer leads to elevated cholesterol production, which serves as a continuous substrate for membrane synthesis in proliferating cells and facilitates the synthesis of estrogen and androgens, thereby promoting tumorigenesis [41, 44].

Tumor cells exhibit a heightened demand for cholesterol due to their rapid multiplication, driving aberrant cholesterol metabolism in OC (OC) and other malignancies [44, 47]. Dysregulated cholesterol biosynthesis, mediated by altered expression of key proteins, supports cancer cell survival, growth, migration, and invasion. This upregulation of cholesterol biosynthesis is a common feature in various cancers and has been linked to poor overall survival in melanoma, acute myeloid leukemia, sarcoma, and breast cancer [48]. Adding to the nutritional and cellular structural needs of cancerous cells that cholesterol fulfills, mevalonate pathway intermediates such as FPP and GGPP are fundamental for protein prenylation and indispensable for regulating mutated signaling molecules such as Ras, Rho, and Rab GTPases, pivotal for cell survival, growth, invasion, and metastasis [32, 45].

OC is one of several malignancies such as prostate, gastric, and colon cancers that have elevated levels of MVA‐related enzymes including HMGCR, SS, SQLE, FDPS, and GGPPS [49, 50, 51, 52]. Studies demonstrate the significance of HMGCS1 and HMGCR in promoting cellular viability and growth, with overexpression of HMGCR promoting cell growth in experimental models and correlating with poor prognosis in breast cancer and OC [50, 53, 54]. A study by Brennan et al. [54] showed that the cytoplasmic expression of HMGCR was present in 65% of OC cancer cases studied using immunohistochemical staining on tissue microarrays. In cancerous cells, HMGCR activity remains resistant to negative feedback control mechanisms, leading to the accumulation of isoprenoids even in environments with high cholesterol levels, thus facilitating the development of malignant tumor features [44, 54, 55, 56].

Cholesterol is well documented as a fundamental metabolic need in cancerous cells [40, 45]. An early nested prospective study conducted by Helzlsouer et al. [57] found a directly proportional correlation between blood cholesterol levels and the risk of OC, specifically with cholesterol levels higher than 200 mg/dL. Further investigations by Li et al. [58] investigated the connection between statins and EOC in a cohort of 132 women with stage III or IV epithelial ovarian or primary peritoneal cancer. Results showed that patients with normal LDL levels exhibited significantly longer median PFS and overall disease‐specific survival compared to those with elevated LDL levels. Additionally, in vivo experiments using an OC mouse model demonstrated that mice fed a high‐cholesterol diet exhibited slightly accelerated tumor growth compared to those on a standard control diet, corroborating clinical observations linking cholesterol levels to OC progression [59].

##### 2.2.3.2 Extracellular Lipid Uptake

Cancer cells augment their lipid profile not only through de novo lipogenesis but also via increased lipid uptake from the extracellular environment [40, 41]. The acquisition of exogenous fatty acids (FAs) also facilitates cell migration and metastasis by altering membrane fluidity. Under stress conditions like hypoxia, where de novo FA synthesis is compromised, cancer cells compensate by absorbing external lipids, orchestrated by the master regulator HIF‐1α and lipid‐binding proteins like fatty acid‐binding protein 4 (FABP4) [38, 40, 41, 60]. Elevated expression of FABP4 has been documented across various tumor types, including OC [41]. This uptake can occur via several pathways, including specialized transport mechanisms involving CD36 fatty acid translocase, fatty acid transport proteins (FATPs) belonging to the SLC27 family of solute carriers, or receptor‐mediated endocytosis of low‐density lipoprotein (LDL) particles through the LDL receptor (LDLR). These transporters are notably upregulated across various cancer types [41]. LDLR, a transmembrane protein crucial for cellular cholesterol uptake, is implicated in chemo‐resistance in OC, with elevated expression correlating with poor prognosis and resistance to platinum‐based chemotherapy [61]. The LDLR/LPC/FAM83B/FGFRs axis mediates this resistance, with SREBP2 possibly regulating LDLR binding [62]. Moreover, CD36, another transmembrane glycoprotein, facilitates the uptake of free FAs and cholesterol and plays essential roles in cancer biology, including antigen presentation, inflammation, and angiogenesis [38]. Ladanyi et al. [63] showed that the inhibition of CD36 resulted in a decreased uptake of fatty acids by OC cells, leading to reduced accumulation of cholesterol and lipid droplets, as well as decreased levels of intracellular reactive oxygen species (ROS). Additionally, the knockdown of CD36 resulted in a reduction in adipocyte‐mediated invasion and migration of cancer cells.

##### 2.2.3.3 Lipid Storage

Heightened de novo lipid synthesis and uptake in cancer cells necessitate the storage of excess lipids, typically in the form of lipid droplets. These droplets are synthesized within the endoplasmic reticulum through the action of sterol O‐acyltransferase 1 (SOAT1), also known as acyl‐CoA acyltransferase 1 (ACAT1) [41, 64]. Compared to normal cells, cancer cells typically exhibit an increased abundance of lipid droplets. Lipid droplets serve multiple crucial functions in cancer cells, including maintaining lipid homeostasis, preventing lipotoxicity, regulating autophagy, preserving endoplasmic reticulum (ER) and membrane homeostasis, and acting as a source of ATP and NADPH upon breakdown via lipophagy followed by β‐oxidation during metabolic stress. Accumulation of lipid droplets is observed in OC with a correlation to enhanced tumorigenesis [65, 66].

##### 2.2.3.4 Activation of Lipolysis and Fatty Acid Oxidation

Tumor cells additionally obtain fatty acids (FAs) by initiating lipolysis, a process involving the breakdown of lipid droplets facilitated by lipoprotein lipase (LPL), resulting in the liberation of free FAs. These liberated FAs are subsequently absorbed by CD36 and utilized to sustain enhanced cellular growth [41]. Lipolysis fuels further degradation via FAO, commonly known as β‐oxidation. The transport of cytoplasmic FAs for FAO at the mitochondria is essential for cancer cell survival and development. β‐oxidation serves as a vital source of ATP and NADPH, providing energy and reducing power for biosynthesis and combating oxidative stress. Moreover, the microenvironment surrounding the tumor profoundly influences FAO dynamics. In OC, for instance, the omentum represents a prominent site of metastasis, characterized by a high abundance of adipocytes that serve as a rich source of lipids. These lipids are utilized by cancer cells as a fuel source, thereby modulating metabolic stress during the metastatic process [40, 41].

##### 2.2.3.5 Initiators of Metabolic Reprogramming

Achieving metabolic plasticity in cancer cells necessitates the induction of a multitude of genes, along with the activation or inhibition of various oncogenes, growth factors, and tumor suppressors. The intricate molecular mechanisms that confer tolerance to prolonged hypoxia and nutrient/energy starvation in cancer cells operate at both transcriptional and post‐translational levels, orchestrated by the Hypoxia‐Inducible Factor (HIF) signaling pathway. These factors are expressed in all eukaryotes through three isoforms: HIF‐1, HIF‐2, and HIF‐3. HIF‐1, a master regulator activated under low oxygen levels, plays a crucial role in modulating cellular metabolism. In instances of acute hypoxia, HIF‐1α forms a dimer with HIF‐1β, leading to its stabilization. Subsequently, it undergoes translocation into the nucleus, where it binds to hypoxia response elements (HREs) on DNA, thereby regulating gene expression. Under hypoxic conditions, HIF‐1 induces the expression of several genes involved in glycolysis and the pentose phosphate pathway (PPP) including the upregulation of glucose transporter (GLUT1), the rate‐limiting glycolytic enzyme hexokinase (HKII), glycolytic regulator phosphofructo‐2‐kinase (PFK1), pyruvate kinase (PK), pyruvate dehydrogenase kinase (PDK), lactate dehydrogenase (LDH), and phosphoglycerate kinase 1 (PGK1) promoting cancer cell survival even in oxygen‐deprived environments. Simultaneously, there exists a concurrent downregulation or occurrence of mutations in genes encoding the pyruvate decarboxylase complex (PDC), enzymes in the tricarboxylic acid (TCA) cycle, or the ETC enzymatic complex I [67]. This critical pathway effectively reroutes glucose metabolism to support cancer cell survival in oxygen‐deprived environments [67]. Additionally, signal transduction pathways activated by growth factors, such as epidermal growth factor (EGF) and platelet‐derived growth factor (PDGF), converge on the Ras/PI3K/phosphatase and tensin homolog (PTEN)/Akt and mTOR signaling pathways, further enhancing HIF‐1 expression and activity. mTOR signaling, a central regulator of cell growth and metabolism, plays a dual role in cancer progression by both activating and being activated by HIF‐1. This positive feedback loop between mTOR and HIF‐1 reinforces the metabolic reprogramming necessary for tumor survival, highlighting the interconnectedness of signaling pathways in cancer metabolism [68]. HIF‐1 also promotes angiogenesis by regulating vascular endothelial growth factor (VEGF), ensuring a continuous nutrient supply to support tumor growth [25, 67]. Simultaneously, the tumor suppressor p53, known for its role in maintaining genomic stability, counteracts the glycolytic shift induced by HIF‐1. p53 promotes oxidative phosphorylation (OXPHOS) and reduces glycolysis by downregulating HKII expression. In OC cells lacking functional p53, this regulatory mechanism is compromised, leading to an intensified reliance on glycolysis for energy production. This metabolic reprogramming, orchestrated by HIF‐1 and influenced by p53 status, creates a microenvironment conducive to tumor growth, progression, and angiogenesis.

Furthermore, an additional layer of complexity in cancer metabolism regulation involves the interplay between AMP‐activated protein kinase (AMPK) and mTOR signaling pathways. AMPK, acting as a cellular energy sensor, becomes activated under conditions of cellular stress, such as nutrient deprivation or hypoxia, inhibiting mTOR signaling to conserve cellular energy and promote catabolic processes. Dysregulation of AMPK and mTOR signaling pathways can profoundly impact tumor growth and progression, making them attractive targets for cancer therapy. This interconnected network of signaling pathways underscores the importance of understanding the role of environmental factors, such as obesity, in cancer metabolism and the development of targeted therapeutic strategies [67].

**FIGURE 3:**
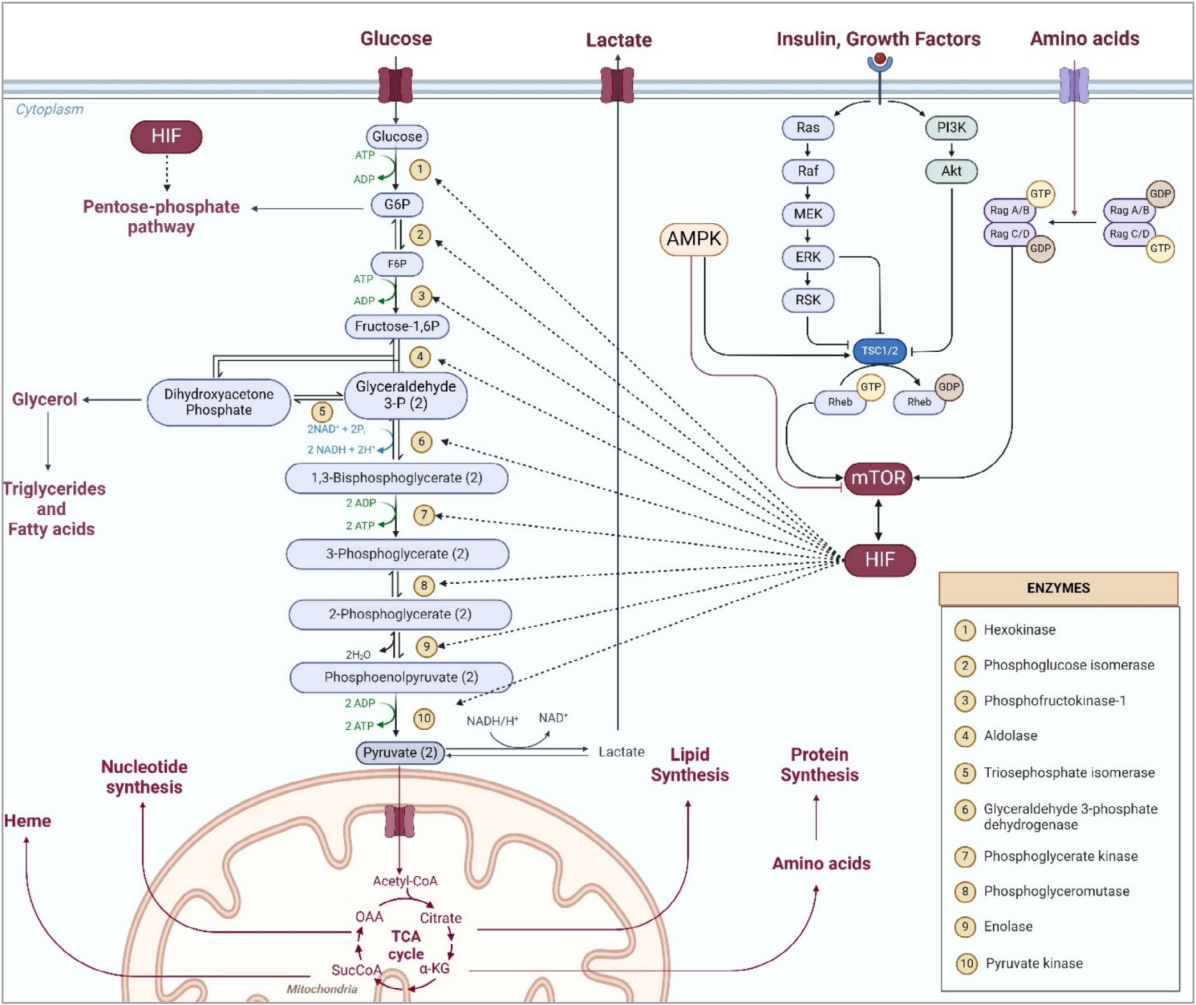
Cancer cellular metabolic reprogramming. This schematic illustrates the cellular metabolic reprogramming in cancer, linking it with obesity and metabolic syndrome, wherein the activation of various components, including the gene encoding glucose transporters, enzymes of the pentose‐phosphate pathway and glycolysis, and pyruvate dehydrogenase complex kinase, is orchestrated by hypoxia‐inducible factor‐1 (HIF‐1), which is influenced by factors including obesity and metabolic syndrome, such as dysregulated mTOR pathway activation and AMPK inhibition.

In the complex interplay between environmental factors and cellular signaling pathways, emerging research suggests a significant role for obesity as a pivotal contributor to cancer progression, elucidating its capacity to drive the activation of HIF‐1α. Hypoxia, hyperinsulinemia, and adipogenesis represent hallmark features of obesity, a condition typified by chronic inflammation and perturbed metabolic states. This activation, instigated by obesity, augments the metabolic reprogramming within cancer cells, propelled by the triad of adipogenesis, insulin perturbations, and hypoxic microenvironments [69]. Understanding these molecular intricacies provides valuable insights into potential therapeutic strategies targeting metabolic vulnerabilities in OC (Figure 3).

#### 2.2.4 Metabolic Plasticity in Ovarian Cancer

Several studies demonstrate that OC cells predominantly adopt a glycolytic metabolism [25]. Key glycolysis markers, GLUT1 and HKII, are significantly upregulated in HGSC compared to non‐HGSC. For instance, the OVCA420 cell line (a serous/non‐CCC type) shows impaired mitochondrial function, reduced oxygen consumption, ATP production, and mitochondrial membrane potential, leading to a metabolic shift toward glycolysis. This shift involves altered mitochondrial morphology and increased fission mediated by dynamin‐related protein 1 (Drp1) [70]. However, evidence indicates that HGSC and CCC can undergo metabolic reprogramming toward OXPHOS to support survival [25]. CCC cell lines like ES‐2 and TOV‐21‐G exhibit high metabolic activity with elevated glycolysis‐related gene expression [70]. CCC cells overexpressing the transcription factor HNF‐1β rely on glycolysis over OXPHOS for energy production, as HNF‐1β promotes glucose uptake and glycolysis [71]. Despite this, EOC, including HGSC and CCC, shows increased overall OXPHOS activity compared to normal cells, with higher mitochondrial respiration markers, such as elevated OXPHOS enzymes and respiratory capacity [72].

These findings highlight that EOC relies on both glycolysis and OXPHOS, demonstrating metabolic flexibility and the ability to shift between pathways. The metabolic profile varies across ovarian cancer subtypes and is influenced by genomic, epigenetic factors, and technical variations [25].

## 3 Therapeutic Advancements: Leveraging Drug Repurposing Strategies to Address Metabolic Plasticity in Ovarian Cancer

In recent years, the landscape of cancer therapeutics has witnessed a paradigm shift, transitioning from traditional approaches of de novo drug development to more innovative strategies such as drug repurposing. This evolution stems from the need for efficient and cost‐effective solutions to combat the complexities of cancer, including OC. OC, characterized by its metabolic plasticity, presents a formidable challenge in treatment, necessitating novel therapeutic approaches. In this context, the convergence of therapeutic advancements and drug repurposing strategies holds immense promise for overcoming the hurdles posed by metabolic plasticity in OC.

### 3.1 De Novo Drug Development

The tremendous expansion in the field of cancer biology, coupled with advancing technology and a deeper understanding of tumorigenesis throughout the cancer hallmarks, is driving the development of novel antineoplastic medications. This progress plays a pivotal role in minimizing cancer death rates. Nevertheless, progress in the domain of drug discovery, specifically in OC, is still slow and hindered by excessive costs and the long timeframe before marketing [73, 74]. Conventional methods for oncological drug discovery take, on average, around 15 years of research and require billions of dollars of groundwork investments. The odds of a new compound candidate progressing from initial research to a phase I trial are merely 10%, with a staggering 1 in 30,000 chances of successfully becoming a new drug. Adding to this, escalating drug development expenses contribute to the growing costs of oncological medications, posing a significant concern for the future of medical research that necessitates consideration of medical economics [73, 75]. This extensive research framework, established in the wake of the thalidomide congenital malformations disaster in the 1960s [76], requires a rigorous four‐phase process for FDA approval [73, 77]. Phase I clinical trials initiate the assessment by studying the safety of the new drug in a small cohort (e.g., 20–80 participants). Phase II studies expand into a bigger number of individuals (few hundred people) [73]. Phase III studies involve a more extensive participant pool (from hundreds to thousands), comparing the novel drug with other interventions and assessing efficacy while tracking side effects. Phase IV trials, or post‐marketing studies, follow the drug after commercialization, tracking its impact and effectiveness in the public and gathering data on long‐term side effects. Despite years of research and even after receiving approval, numerous newly discovered therapies face challenges due to high toxicity rates, leading to subsequent failures in clinical application.

### 3.2 Drug Repurposing Strategy

#### 3.2.1 Definition

The adoption of the Drug repurposing strategy (DRS) also known as drug reprofiling, repositioning, or re‐tasking, stands as an important approach in cancer management. This tactic involves the redirection of previously approved drugs with known pharmacokinetic and pharmacodynamic characteristics for alternative indications. Leveraging the known toxicity profiles of approved drugs in diverse pathologies significantly reduces infrastructure costs and research timelines while ensuring patient safety remains uncompromised [78, 79]. The rationale behind DRS is that molecules with low molecular weight would most likely target more than just one of the numerous proteins found in the human body [80]. It is worth noting that the two major bodies for pharmaceutical control and regulation, the Food and Drug Administration and the European Medicines Agency, have introduced drug repurposing projects to direct the reprofiling of clinically approved and marketed medications [81].

#### 3.2.2 DRS Vs. De Novo Drug Development

**FIGURE 4:**
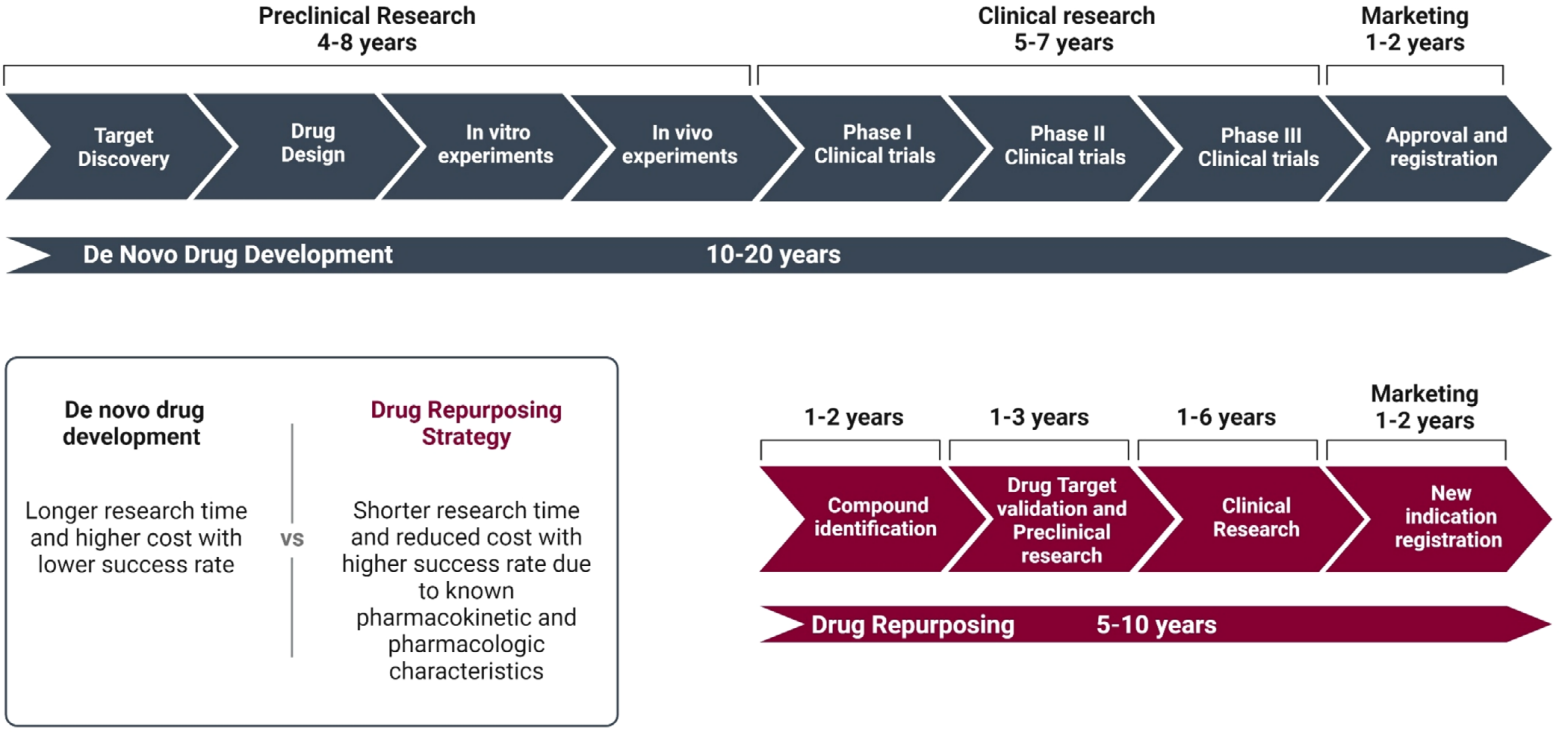
De novo drug development versus Drug Repurposing. Schematic comparison between the processes and timeline of de novo drug development and drug repurposing.

When compared to de novo drug development, this approach offers several advantages. First and foremost, the repurposed therapeutic agent is less likely to encounter setbacks in later efficacy trials, given its prior establishment of safety through preclinical and clinical models. This is possibly the most crucial benefit of all. Second, because most preclinical studies and toxicity evaluations will be already completed, the timeline for drug development can be shortened (Figure 4). Third, less investment is required, though this may vary substantially depending on the reprofiled agent [82]. Finally, this strategy capitalizes on therapeutic agents that have already undergone clinical evaluation, providing a more comprehensive understanding of the drug's pharmacokinetic, pharmacologic, and biomolecular mechanisms [83].

### 3.3 Overcoming Metabolic Plasticity in Ovarian Cancer by Drug Repurposing Strategy

**FIGURE 5:**
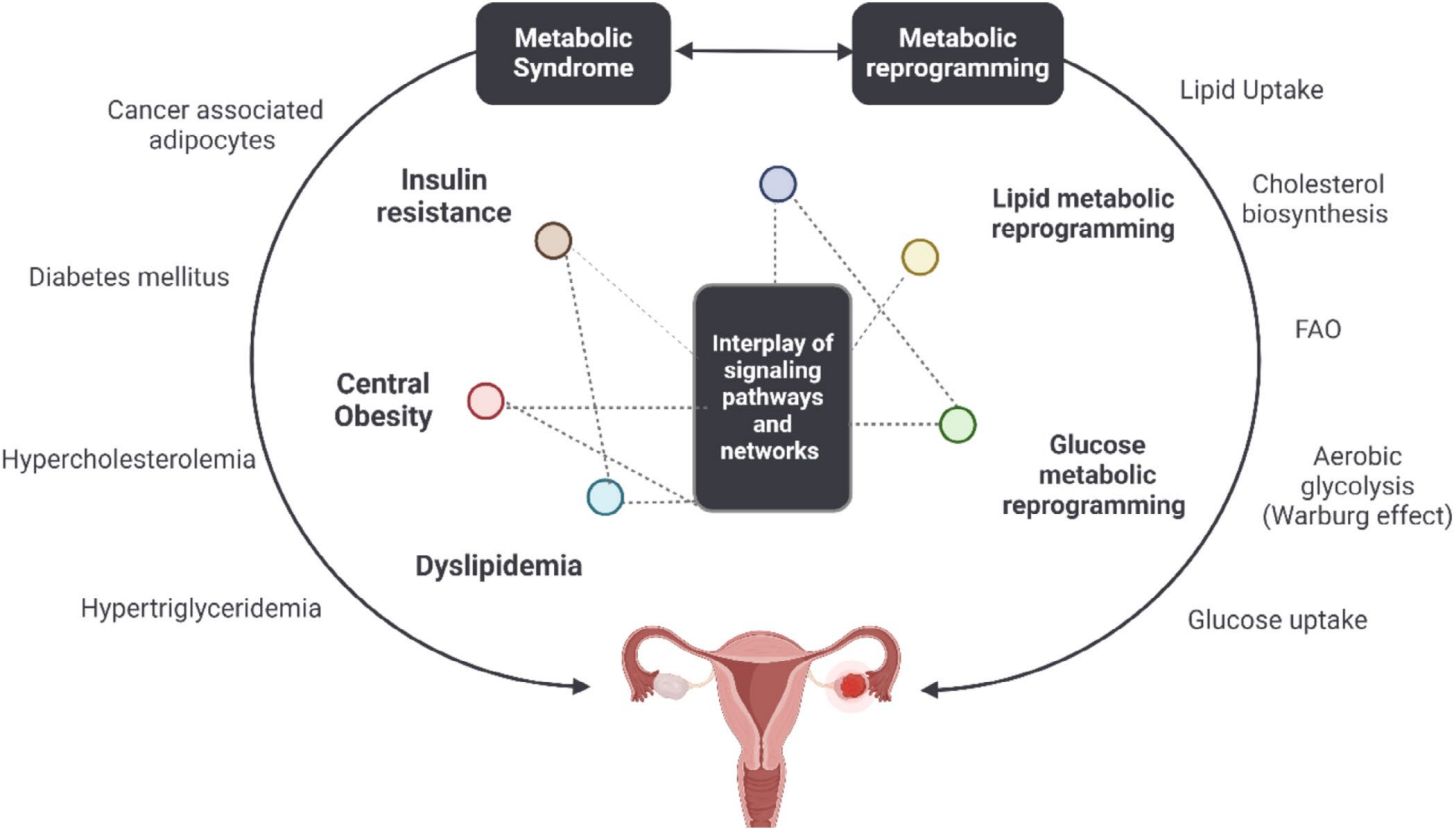
Interplay between metabolic syndrome and cellular metabolic reprogramming. The metabolic syndrome includes Obesity, Diabetes mellitus and dyslipidemia that in turn are the result of several factors including hyperglycemia, hypercholesterolemia, insulin resistance, and chronic inflammation. Parallelly, any changes in lipogenesis, FAO, cholesterol biosynthesis, glycolysis, lipid, and glucose uptake lead to a dysregulation in cellular metabolism. The interaction between both pathways and networks is essential for OC progression.

The recognition and comprehensive comprehension of cellular metabolic reprogramming and the metabolic syndrome as pivotal orchestrators of cancer initiation and advancement have underscored the rationale behind leveraging repurposed metabolic‐targeting medications in OC (Figure 5). Among these medications are metformin and statins, which have garnered attention for their potential therapeutic benefits beyond their original clinical indications [23, 84].

#### 3.3.1 Metformin

##### 3.3.1.1 Overview and Pharmacotherapeutic Effects

Metformin (MTF) is a biguanide, a synthetic derivative of guanidine, the active component of the French Lilac, which was originally prescribed for the treatment of polyuria during the Middle Ages. However, concerns regarding lactic acidosis and cardiovascular mortality delayed its approval as an oral anti‐diabetic medication in the USA until 1995. Fortunately, extensive safety assessments over time have affirmed the well‐tolerated nature of metformin [85]. A comprehensive Cochrane evaluation, incorporating data from 347 cohort studies and comparative trials, found no evidence of elevated lactic acidosis or lactate levels compared to other anti‐diabetic agents [86].

MTF has since become a cornerstone in the management of type II diabetes (T2D), owing to its robust efficacy in lowering blood sugar levels, ensuring glycemic control, its established safety profile, and its affordability [87]. Primarily acting on the liver, metformin enhances insulin sensitivity and curtails hepatic glucose production (HGP) by inhibiting hepatic gluconeogenesis, consequently reducing insulin and glucose levels. Furthermore, it facilitates glucose uptake and utilization in skeletal muscle and adipose tissue, albeit to a lesser extent [88, 89].

The predominant mechanism underlying metformin's glucose‐lowering effects revolves around the suppression of complex I activity within the mitochondrial electron transport pathway upon cellular entry. This leads to a decline in cellular adenosine triphosphate (ATP) levels and an elevation in the adenosine monophosphate/ATP ratio. While the precise mode of this suppression has long puzzled researchers, it is now understood that metformin may activate AMP‐activated protein kinase (AMPK), a key cellular energy sensor. Activation of AMPK occurs via phosphorylation at the Thr172 residue by calcium/calmodulin‐dependent protein kinase (CaMKK) and liver‐kinase b1 (LKB1), shifting cellular metabolism from an anabolic to a catabolic state. Consequently, gluconeogenic gene expression is downregulated, and there is a reduction in lipid, protein, and glucose synthesis, along with heightened insulin sensitivity and enhanced cellular uptake of glucose and fatty acids [85, 87].

The health benefits of MTF have grown over time. Besides blood sugar control, metformin is also prescribed to treat certain metabolic disorders such as polycystic ovarian syndrome (PCOS) [90] and gestational diabetes [91]. Additionally, metformin shows protective effects against numerous additional age‐related morbidities such as cardiovascular disease, and cognitive decline [92].

##### 3.3.1.2 Effects of Metformin on the Risk of OC

Numerous pivotal studies have spurred investigations into the mechanistic actions of MTF in OC. Notably, Kumar et al. [93] conducted a retrospective case–control study comparing OC patients treated with metformin (cases) to those untreated (controls). The analysis revealed significantly improved survival rates among cases compared to controls (*p* = 0.0002), indicating a potential association between metformin intake and enhanced survival outcomes in OC.

##### 3.3.1.3 Mechanism of Action of Metformin in Cancer

In 2005, Scottish researchers conducted an observational study revealing that patients with Type 2 Diabetes (T2D) who took MTF exhibited a lower cancer risk. This groundbreaking discovery suggested, for the first time, the potential antitumor properties of MTF [94]. Since then, it has garnered significant attention as a potential anti‐cancer drug, prompting researchers worldwide to investigate its mechanism of action.

Broadly, MTF functions by restoring metabolic homeostasis, which is often dysregulated in cancer cells. As previously mentioned, some of the most striking changes in tumor cellular bioenergetics include the elevation of aerobic glycolysis (Warburg effect), an increase in lipid and protein synthesis, overexpression of glucose transporters, and an increase in mitochondrial biogenesis. Remarkably, MTF has demonstrated the ability to target the previously discussed glucose metabolic reprogramming, ultimately inducing apoptosis and inhibiting cellular proliferation [95]. To achieve these effects, MTF operates through both direct and indirect mechanisms, as described previously [85].

**FIGURE 6:**
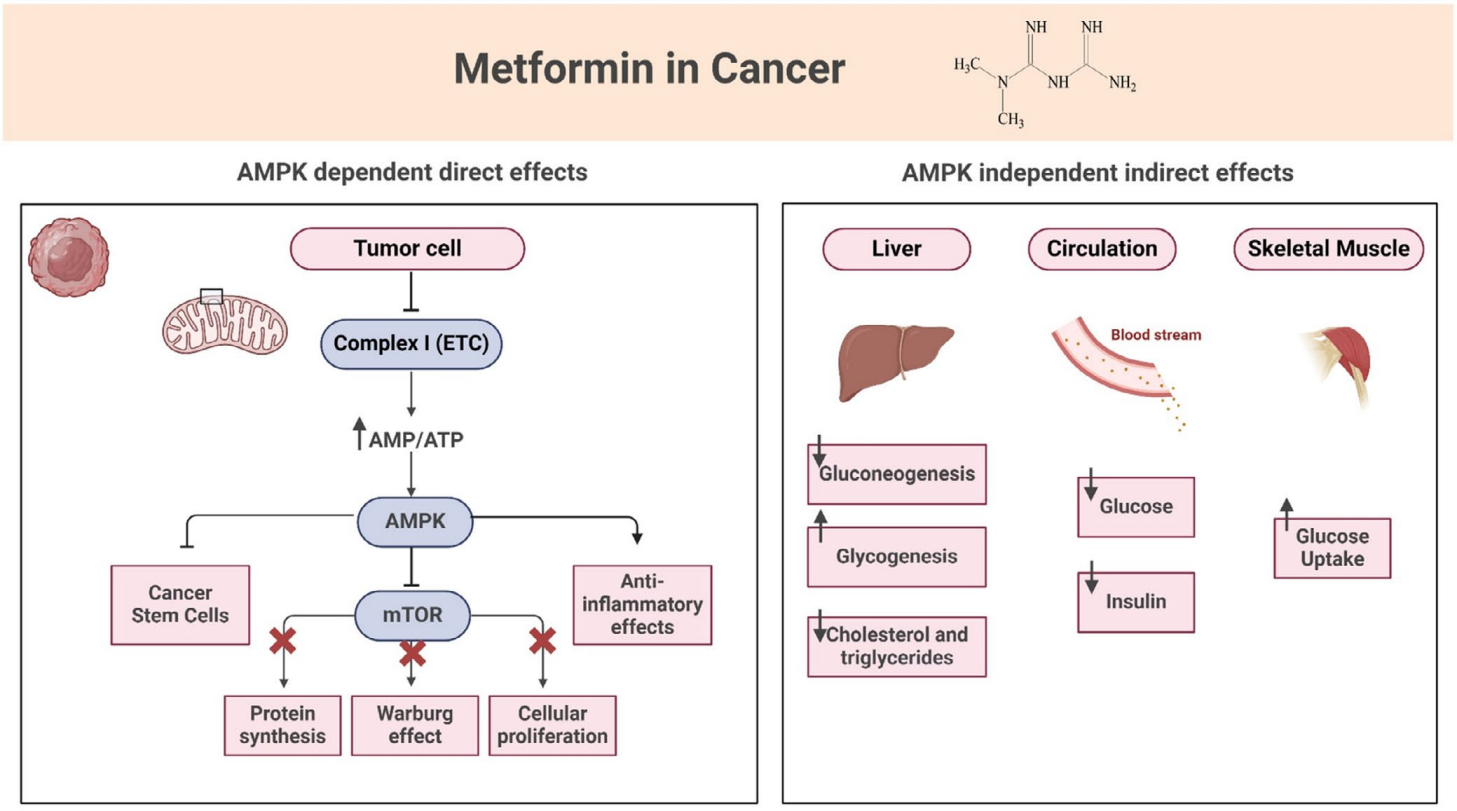
Direct and indirect effects of metformin in cancer. Metformin possesses both direct (Left) (AMPK dependent) and indirect (right) (AMPK independent) effects on a multitude of pathways and networks.

In the AMPK‐dependent pathway, MTF initiates its action by inhibiting complex I, a mitochondrial electron transport protein. This inhibition disrupts mitochondrial ATP production, resulting in an increase in the AMP/ATP ratio and a subsequent decrease in hepatic energy status. The reduced energy state of the liver leads to the binding of AMP to AMPK, enhancing its affinity for liver serine–threonine kinase B1 (LKB1). Consequently, the activated AMPK‐LKB1 complex inhibits the AKT/mTOR network signaling pathway [89]. Furthermore, MTF's inhibition of mTOR extends to other cancer‐promoting pathways, including the downregulation of c‐Myc [96], nuclear transcription factor κB (NF‐κB) [97], and the p53 family proteins [98], as illustrated in Figure 6. AMPK activation also appears to counteract the metabolic shift toward the Warburg effect phenomenon, a hallmark of many cancer cells characterized by increased aerobic glycolysis even in the presence of oxygen. Moreover, AMPK activation exhibits an anti‐inflammatory effect by reducing the production of pro‐inflammatory cytokines such as interleukin‐6 and 8 (IL‐6, IL‐8), and vascular endothelial growth factor (VEGF). Since IL‐6 production stimulates the signal transducer and activator of transcription protein (STAT), which promotes cell proliferation, inhibiting IL‐6 can attenuate cellular proliferation. Additionally, AMPK activation may play a crucial role in suppressing the continual regeneration of cancer stem cells (CSCs), which are often resistant to chemotherapy and radiotherapy. MTF enhances the response to chemotherapy by targeting CSCs in various cancers, potentially through AMPK activation and mTOR suppression. Furthermore, MTF exhibits an immunomodulatory effect on cancer cells by stimulating CD8+ tumor‐infiltrating lymphocytes (TILs), leading to a cytotoxic response against cancer cells. Lastly, MTF appears to protect against DNA damage by inhibiting reactive oxygen species and activating Ataxia Telangiectasia Mutation (ATM), a critical component of DNA repair mechanisms. Its ability to prevent DNA damage contributes to its overall anticancer properties [85].

In addition to the AMPK‐dependent pathway, MTF exerts its anticancer effects through an AMPK‐independent pathway. In this pathway, MTF reduces circulating insulin levels and inhibits insulin and insulin‐like growth factor (IGF‐1) signaling. Normally, under nutrient‐rich circumstances, IGF‐1 binds to the IGF‐1 receptor (IGF‐1R), leading to the activation of the PI3K/AKT/mTOR and RAS/fibrosarcoma rapid acceleration (RAF)/MAPK signaling pathways. Activation of these pathways enhances cell proliferation and promotes signaling pathways associated with cancer aggressiveness. By decreasing circulating insulin, MTF interferes with IGF‐1/IGF‐1R signaling and inhibits the PI3K and MAPK signaling pathways (207). This inhibition ultimately suppresses cell proliferation and reduces the aggressiveness of cancer cells. Moreover, MTF's ability to modulate insulin and IGF‐1 signaling contributes to its role in metabolic regulation and may have implications for cancer prevention and treatment [87]. Furthermore, emerging evidence suggests that MTF's effects on insulin and IGF‐1 signaling extend beyond cancer cells to impact the tumor microenvironment. MTF's modulation of insulin and IGF‐1 signaling may influence the interactions between cancer cells and surrounding stromal cells, immune cells, and blood vessels within the tumor microenvironment. Moreover, MTF decreases gluconeogenesis and increases glycogenesis in the liver, while also decreasing cholesterol and triglyceride production. In skeletal muscle, it increases glucose uptake, thereby decreasing circulating glucose levels [97].

Overall, MTF's dual effects on AMPK‐dependent and AMPK‐independent pathways highlight its complex mechanism of action and its potential as a promising therapeutic option for cancer prevention and treatment (Figure 6) [85].

##### 3.3.1.4 Effects of Metformin on Ovarian Cancer—Preclinical Evidence

Early in vitro studies conducted by Gotlieb et al. [99] demonstrated that MTF inhibited the growth of OC cells in a dose‐ and time‐dependent manner, mediated through AMPK‐dependent pathways. Subsequent research by Rattan et al. [100] further elucidated MTF's effects, revealing significant inhibition of proliferation in various OC cell lines with differing chemotherapeutic sensitivities, involving both AMPK‐dependent and independent pathways. Additionally, this study revealed that MTF induced cell cycle arrest, accompanied by decreased cyclin D1 levels and increased expression of p21 protein. Moreover, Erices et al. [101] found that MTF potentiates the cytotoxic effects of carboplatin on two distinct OC cell lines. Table 1 provides a summary of additional preclinical studies exploring the efficacy of MTF.

**TABLE 1:** Preclinical evidence conducted with MTF in ovarian cancer models.

Study by	Setting	Results
Shank et al. [102]	In vitro and in vivo OC models treated with MTF	MTF inhibited the development, angiogenesis, and proliferation of cancer stem cells.
Kim et al. [103]	SK‐OV‐3 cells treated with MTF	After MTF treatment, Axl and Tyro3 were inhibited, while Erk and STAT3 were activated.
Patel et al. [104]	Primary human OC cells treated with MTF	MTF elicited apoptosis and cell cycle arrest with the BCL‐2 family playing a critical role
Zou et al. [105]	SK‐OV‐3 cells treated with MTF	MTF inhibited invasion, migration, and proliferation.
Ma et al. [106]	In vitro and in vivo OC models treated with MTF	MTF cytotoxicity was glucose‐dependent and mediated by ASK‐1.

##### 3.3.1.5 Effects of Metformin in Ovarian Cancer—Clinical Trials

Currently, there are three completed clinical trials investigating the use of MTF for OC, with one trial having published its results and two others actively recruiting participants [107]. The published phase II clinical trial analyzed MTF's impact on carcinoma‐associated mesenchymal stem cells (CA‐MSCs), cancer stem cells (CSCs), and clinical outcomes in non‐diabetic patients with advanced epithelial OC (EOC). This trial demonstrated that MTF altered the epigenetic methylation profile in CA‐MSCs, thereby enhancing sensitivity to cisplatin ex vivo. Additionally, MTF therapy was associated with better‐than‐expected overall survival, supporting the use of MTF in phase III studies [108].

#### 3.3.2 Statins

##### 3.3.2.1 Overview and Pharmacotherapeutic Effects of Statins

Among the array of drugs being investigated in cancer research, statins have emerged as notable candidates. Statins competitively inhibit 3‐Hydroxy‐3‐Methyl‐Glutaryl‐Coenzyme A Reductase (HMGCR), a pivotal enzyme catalyzing the conversion of HMG‐CoA to mevalonic acid. Widely utilized for treating hypercholesterolemia and preventing cardiovascular diseases, statins exhibit multifaceted functions, encompassing inflammation reduction and modulation of vascular expansion through coagulation and fibrinolysis [109, 110, 111, 112].

**FIGURE 7:**
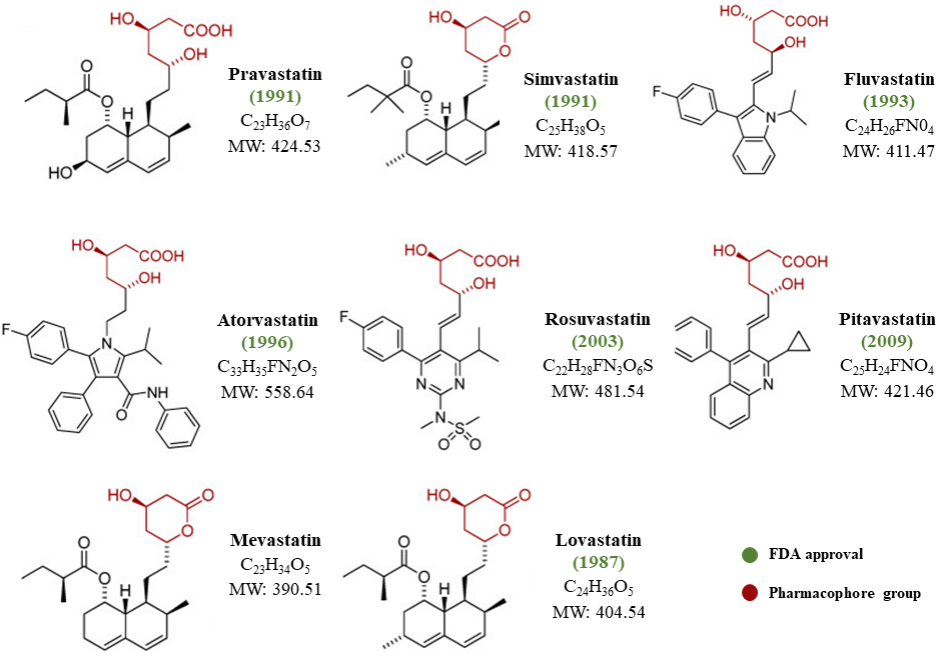
Chemical structure of statins. The first two well‐known statins, vastatin (compactin) and lovastatin (mevinolin), marked significant milestones in the history of cholesterol‐lowering drugs. Mevastatin and lovastatin, both naturally occurring statins, were the first to be isolated. Pravastatin derives from mevastatin, while simvastatin is a semi‐synthetic analog of lovastatin. On the other hand, fluvastatin, atorvastatin, rosuvastatin, and pitavastatin are entirely synthetic statins. All statins, except mevastatin, have obtained approval for human use. A pivotal structural feature shared by all statins is the pharmacophore group. This common structural element serves as the cornerstone of their inhibitory action on 3‐Hydroxy‐3‐Methyl‐Glutaryl‐Coenzyme A Reductase (HMGCR), a critical enzyme in cholesterol biosynthesis [113].

The inception of statins can be traced back to ML‐236B, commonly known as compactin or Mevastatin, which was identified as the first inhibitor of HMG CoA reductase derived from *Penicillium citrinum*. However, due to undesirable side effects observed in animal studies, extensive efforts were made to isolate more effective inhibitors. Lovastatin, or mevinolin, emerged from this pursuit and was discovered in *Aspergillus terreus* in the late 1970s. Upon evaluation in animal models, lovastatin demonstrated superior potency to mevastatin. In 1987, lovastatin became the pioneering statin to enter clinical practice. Since then, a multitude of statin inhibitors have received human approval, including simvastatin, pravastatin, rosuvastatin, fluvastatin, Atorvastatin, and Pitavastatin (Figure 7) [113, 114].

The pharmacophore group, a structural motif shared by all statins closely resembling the HMG‐CoA molecule, endows statins with highly efficient competitive inhibition of HMG‐CoA reductase, exhibiting a 1000‐fold greater affinity to the enzyme compared to the natural substrate [115]. Although possessing closely related pharmacokinetic properties, each statin is unique. Statins operate by diminishing intracellular cholesterol levels through targeted inhibition of HMG‐CoA reductase, thereby impeding de novo cholesterol biosynthesis and reducing hepatic cholesterol levels. This prompts liver cell membranes to upregulate LDL‐receptors (LDL‐R), facilitating the clearance of LDL particles from the bloodstream [114, 115].

##### 3.3.2.2 Effects of Statins on the Risk of Cancer

The current body of research on the effects of statins on OC yields conflicting results. A recent meta‐analysis conducted by Wang et al. [116] analyzed data from 19 studies involving approximately 2 million females, of whom 8000 were diagnosed with OC. The analysis revealed that statin use did not significantly reduce the risk of OC (relative risk (RR) = 0.88, 95% confidence interval (CI) 0.76–1.03, *p* = 0.12). Furthermore, examining statins use over a period exceeding five years showed no discernible evidence of risk reduction for OC (RR = 0.73, 95% CI 0.51–1.04, *p* = 0.08).

However, other studies and meta‐analyses have reported a significant positive impact of statins on OC [117, 118, 119, 120, 121, 122]. According to a meta‐analysis comprising 55 articles [123], statin use demonstrated potential in improving cancer patient survival rates. This comprehensive analysis, involving over a million individuals with more than 18 cancer types, revealed consistent associations between statin use and reduced overall mortality, cancer‐related mortality, recurrence‐free survival, progression‐free survival, and disease‐free survival. Mei et al. [123] found that these associations remained robust across various factors, including study design, sample size, tumor location, statin initiation, disease stage, follow‐up duration, and geographical location of research or participating hospitals. Table 2 provides an overview of additional studies examining the relationship between statin use and risk reduction, as well as enhanced survival outcomes in OC.

**TABLE 2:** Impact of Statins on the risk of ovarian cancer.

Study type	Findings in statin use group	Results
Meta analysis [119]	Decrease in mortality but no association with risk	RR = 0.92, 95% CI 0.85 to 1.00; OS: HR = 0.78, 95% CI 0.73 to 0.83
Meta analysis [124]	Better overall survival and decrease in cancer related mortalities with post diagnostic use of statins	Mortality: HR = 0.74, 95% CI 0.63 to 0.87; cancer‐specific mortality HR = 0.87, 95% CI 0.80 to 0.95.
Meta analysis [125]	Improved survival for statin user's vs. non users	HR = 0.76, 95% CI 0.68–0.85
Cohort study of 126 patients [126]	Improved survival rate among statin user's vs. non users	HR = 0.45, 95% CI 0.23–0.88
Retrospective population‐based study of 5416 patients [121]	Reduced risk of overall mortality and cancer related mortality with post diagnostic use of statins (simvastatin and rosuvastatin)	Mortality (Simvastatin) HR = 0.86, 95% CI:0.74–0.99

##### 3.3.2.3 Mechanism of Action of Statins in Cancer

The burgeoning field of oncology has cast a spotlight on statins, prompted by their potential to counteract the metabolic reprogramming observed in cancer, notably the upsurge in lipid synthesis. When discussing alterations in lipid synthesis, it is essential to consider both fatty acids and cholesterol synthesis. In the context of rapidly proliferating cancer cells, elevated cholesterol synthesis via the mevalonate pathway is imperative for generating membranes crucial for survival and proliferation [115].

As previously mentioned, statins exert their effects by inhibiting the enzyme HMG‐CoA reductase. This inhibition not only reduces mevalonate levels but also diminishes the production of its downstream products, which play pivotal roles as regulators of the cell cycle and modulators of signaling pathways [115]. Results from a study conducted by Fujiwara et al. [127] demonstrated that statins induce G1 cell‐cycle arrest by inhibiting the Ras pathways, augmenting caspase‐9 and caspase‐3 activation, and enhancing Bim expression, ultimately leading to apoptosis. Further research has suggested that statins impede cancer cell proliferation by downregulating the expression of key proteins such as c‐Myc, Ras, and Rho, or by inducing cellular senescence [128].

##### 3.3.2.4 Effects of Statins in Ovarian Cancer—Preclinical Evidence

A study by Kato et al. [129] revealed that OC, endometrial cancer, and cervical cancer cell lines express elevated levels of HMG‐CoA reductase. Additionally, the study demonstrated that both lovastatin and simvastatin, classified as lipophilic statins, induced apoptosis in a time‐and dose‐dependent manner in all three cell lines, while pravastatin, a hydrophilic statin, did not elicit similar effects. Specifically, lovastatin and simvastatin activated caspases 8 and 9, initiating apoptosis. Simvastatin additionally exhibited significant inhibition of HMG‐CoA reductase activity and suppressed the MAPK and mTOR pathways in OC cells, leading to cell cycle arrest, apoptosis, and cellular stress. Furthermore, simvastatin induced DNA damage, decreased cell attachment and invasion, and in in vivo studies, inhibited ovarian tumor development. This inhibition was accompanied by a reduction in the expression of proteins associated with cell proliferation and survival, including Ki‐67, HMG‐CoA reductase, phosphorylated‐Akt, and phosphorylated‐p42/44 [130]. In another study, Greenaway et al. [131] employed an orthotopic syngeneic mouse model of serous OC with mutations in brca1, p53, and Rb proteins. They administered intraperitoneal simvastatin daily and observed significant regression of advanced‐stage tumors and the death of metastatic cells.

##### 3.3.2.5 Effects of Statins in Ovarian Cancer—Clinical Trials

Clinical evidence supports the potential of statins in ovarian cancer treatment. Fourteen clinical trials have explored various aspects, including statins' role in reducing ovarian cancer risk, enhancing chemotherapy efficacy, and preventing recurrence. These trials also investigate statins' combination with therapies such as PARP inhibitors, immunotherapy, and targeted therapies. Additionally, multiple studies assessed statins' impact on biomarkers [132], metastasis [133, 134], immune response [135], and long‐term survival in ovarian cancer patients [54, 136]. Although most trials are ongoing and comprehensive published data are limited, some initial findings suggest that statins might improve chemotherapy efficacy and reduce recurrence, impacting biomarkers and immune responses favorably [137].

## 4 Conclusion and Future Perspectives

The intricate interplay between cellular metabolic reprogramming, metabolic syndrome, and cancer initiation and progression underscores the rationale for exploring repurposed metabolic‐targeting medications in OC. Herein, we highlight the critical pathways by which OC cells rewire their metabolism including enhanced glycolysis and altered lipid metabolism. Understanding the mechanisms of metabolic alterations provides new avenues for therapeutic intervention.

Targeting metabolic pathways presents a compelling strategy for overcoming challenges in standard chemotherapeutic medications and in improving patient outcomes. Metformin and statins, originally designed for other clinical purposes, have emerged as promising therapeutic candidates due to their potential to target metabolic pathways implicated in cancer. However, the clinical translation of metabolic therapies necessitates a deeper understanding of the tumor microenvironment and the metabolic crosstalk between cancer cells and stromal components. Personalized approaches, taking into account the unique metabolic profiles of individual tumors, will be essential for optimizing therapeutic strategies.

Future research should focus on unraveling the complex metabolic networks within ovarian tumors and developing novel agents that can selectively target these pathways. Additionally, clinical trials are needed to validate the efficacy and safety of metabolic therapies in combination with standard treatments. Moreover, emerging evidence suggests that glucagon‐like peptide‐1 receptor (GLP‐1R) agonists, including semaglutide and liraglutide, hold a dual role in cancer biology [138]. While semaglutide shows promise in reducing tumor burden in preclinical models of NASH‐induced hepatocellular carcinoma [139], liraglutide's effects are more complex and context‐dependent. Preclinical and clinical studies highlight its potential as both an epigenetic modulator with antitumoral properties and as a promoter of cancer progression under certain conditions, such as triple‐negative breast cancer [140]. Eftekhari et al. [141] investigated the synergistic effects of Liraglutide in combination with Docetaxel on LNCaP prostate cancer cells. Their findings revealed a significant reduction in cell viability and an increase in apoptosis, mediated through suppressed phosphorylation of ERK1/2 and AKT, key regulators of the ERK/MAPK and PI3K/AKT pathways. This synergistic interaction allowed for a reduced Docetaxel dose, suggesting a strategy to mitigate its associated toxicities and resistance. However, conflicting evidence highlights a potential pro‐tumorigenic role under specific conditions, such as enhancing progression in triple‐negative breast cancer via NOX4/ROS/VEGF pathways [142]. Future research should focus on elucidating the molecular mechanisms underpinning these divergent effects, identifying patient subpopulations who may benefit or be at risk, and exploring potential combinatorial strategies to maximize therapeutic efficacy while minimizing oncogenic risks. Additionally, rigorous long‐term studies are required to confirm the safety profile of these agents in populations at risk for cancer.

In summary, metabolic reprogramming in ovarian cancer is not only a hallmark of tumor progression but also a fertile ground for therapeutic innovation. The ongoing advancements in our understanding of cancer metabolism promise to open new frontiers in the fight against ovarian cancer, offering hope for more effective and targeted treatments in the future.

## Author Contributions

Sara Mikhael contributed to the conception, design, acquisition, and analysis of data, and she contributed to the writing of the review manuscript. Georges Daoud contributed to the conception, design, and writing of the manuscript.

## Ethics Statement

The authors have nothing to report.

## Conflicts of Interest

The authors declare no conflicts of interest.

## Data Availability

The authors have nothing to report.
